# Lipidomic dataset of plasma from patients infected with wild type and nef-deficient HIV-1 strain

**DOI:** 10.1016/j.dib.2015.11.067

**Published:** 2015-12-11

**Authors:** Peter Meikle, Hann Low, Melissa J. Churchill, Michael Bukrinsky, Dmitri Sviridov

**Affiliations:** a Baker IDI Heart and Diabetes Institute, PO Box 6492, Melbourne, VIC 3004, Australia; b Macfarlane Burnett Institute for Medical Research and Public Health, 85 Commercial Rd, Melbourne, VIC 3004, Australia; c Department of Microbiology, Immunology and Tropical Medicine, George Washington University, 2300 I St. NW, Ross Hall, Washington DC 20037, USA

## Abstract

Previous in vitro and in vivo studies demonstrated that HIV protein nef plays a key role in impairing cellular and systemic cholesterol metabolism in HIV disease, but clinical support for these findings is lacking. Here we present the data of comparative lipidomic analysis (330 lipid species) of plasma samples from HIV-negative subjects, patients infected with WT HIV-1 strain and patients infected with nef-deficient strain of HIV-1. We determine which effects of HIV on plasma lipidome are explained by the presence of nef. The data can be used to evaluate cardiovascular risk in HIV disease and to assess the role of nef in HIV-induced disturbances in systemic lipid metabolism. The full impact of nef deficiency on lipid and lipoprotein metabolism in HIV-infected patients is presented in the accompanying study “Lipid Metabolism in Patients Infected with Nef-deficient HIV-1 Strain” [1].

Specifications Table

**Table:** 

Subject area	*Medicine*
More specific subject area	*Infectious diseases/HIV*
Type of data	*Table*
How data was acquired	*Mass spectroscopy, Agilent 1200 liquid chromatography system, and Applied Biosystems API 4000 Q/TRAP mass spectrometer*
Data format	*Means*±*standard deviations*
Experimental factors	*Patients infected with wild type and nef-deficient HIV-1 strain*
Experimental features	*Lipids from 10 μl of plasma were extracted using a modified, single phase Folch method and lipidomic analysis was performed by liquid chromatography electrospray ionization-tandem mass spectrometry*
Data source location	*Melbourne, Australia*
Data accessibility	*Data is with this article*

Value of the data

• The data can be used to assess the effect of HIV disease on lipid metabolism.
• The data can be used to assess the role of HIV nef in modifications of lipid metabolism in patients with HIV disease.
• The data can be used to evaluate cardiovascular risk in HIV disease.

## 1 Data

Table 1 shows concentrations of the individual lipid species (lipidomic analysis) in plasma samples from HIV-negative subjects, patients infected with WT HIV-1 strain and patients infected with nef-deficient strain of HIV-1.

## 2 Experimental design, materials and methods

Patients infected with nef-deficient strain of HIV-1 (ΔNefHIV, *n*=6) were all members of the SBBC cohort. They were compared with patients infected with Nef-positive (WT) HIV-1 strain (*n*=6) and six HIV negative subjects selected from a group of healthy volunteers. Description of the patients is presented in the main paper [1].

Lipidomic analysis was performed as described previously [2]. In brief, lipids from 10 μl of plasma were extracted using a modified, single phase Folch method; the analysis was done in triplicates. The analysis was performed by liquid chromatography electrospray ionization-tandem mass spectrometry (LC ESI-MS/MS) using a Agilent 1200 liquid chromatography system, and Applied Biosystems API 4000Q/TRAP mass spectrometer with a turbo-ion spray source (350 °C) and Analyst 1.5 and MultiQuant data systems using a Zorbax C18, 1.8 μm, 50×2.1-mm^2^ column (Agilent Technologies). Lipid concentrations were calculated by relating the peak area of each species to the peak area of the corresponding internal standard.

## Figures and Tables

**Table 1:** Abundance of lipid species in patient plasma.

**Lipid Species**	**HIV Negative**	**WT HIV**	**HIV (∆Nef)**
dhCer 16:0	46.3±6.6	71.5±15.7⁎	120.7±70.4⁎⁎
dhCer 18:0	46.1±11.3	79.5±22⁎	194.1±184.3⁎
dhCer 20:0	20.8±5.6	23.4±9.1	115.7±125.8⁎
dhCer 22:0	89.9±18.6	125±44.4	274.2±187.3
dhCer 24:0	207.2±42.7	260.1±98.7	470.1±288.5
dhCer 24:1	108.6±31	153±42.8	264.1±197.2
Cer 16:0	216.9±20.5	246.3±55.8	344.7±171
Cer 18:0	80.4±16	102.9±26.6	140.7±76.9⁎
Cer 20:0	84.7±17.8	86.2±15.4	166.2±122.2
Cer 22:0	663.2±67.8	617.6±84.4	723.6±266.5
Cer 24:0	2647.7±320.9	2155.3±350.6	2014.1±684.1
Cer 24:1	1089.8±474.5	963.3±253.2	862±225.7
MHC 16:0	1285.4±308	1618.9±471.6	1402.1±398.4
MHC 18:0	252±77.1	231.8±41.4	270.3±67.3
MHC 20:0	259.7±83.5	230.8±48.8	278.3±106.6
MHC 22:0	2758.9±769.8	2474.4±705.9	2608.6±1102.7
MHC 24:0	3975.1±1330.2	3863.1±1217.4	3821.1±1177.1
MHC 24:1	3203.8±1586.7	3114.6±883.1	2508.1±899.7
DHC 16:0	6594±1387	5239.7±1178.6	5250.8±1168.1
DHC 18:0	117.6±20.7	115.1±26.7	159.7±79.3
DHC 20:0	111.2±38.3	85±38.6	256.2±249.5#
DHC 22:0	520.3±182.7	368.2±118.4	1128.3±1010.9
DHC 24:0	540.2±183.4	373.3±111	888±659.1
DHC 24:1	2043.7±630.5	1372.2±370.9	1522.4±534.9
THC 16:0	1250.3±153.9	1252±311.6	1344.5±437.2
THC 18:0	203.2±49.4	178.9±56.8	257.3±106.9
THC 20:0	67.1±6.5	55.7±15.9	139.3±116
THC 22:0	289.5±41	243.6±49	687.3±694.6
THC 24:0	350.7±54.1	301.5±68.5	615.5±505.2
THC 24:1	698.6±71	643.6±166.5	953.8±653.3
GM3 16:0	1186.8±142.4	1499.7±503.6	1250.2±314.1
GM3 18:0	794.1±125.8	748.6±195.3	791.6±191.6
GM3 20:0	361.1±94.8	353.9±72.9	444.7±134.2
GM3 22:0	1177±273.1	1137.9±223.4	1274.8±443.1
GM3 24:0	1179.9±286.4	928.2±204	1012.7±180.2
GM3 24:1	1918.4±1014.5	1692±532.3	1581.7±471.8
SM 31:1	347±110.5	316.1±60.8	385.2±149.5
SM 32:0	423±84.8	512±136.4	666.2±176⁎
SM 32:1	10970.2±2867.3	11084.2±1986.6	12645.4±3860.1
SM 32:2	868.4±175.4	828.5±196.6	961.5±461
SM 33:1	6931.2±1781.1	7052.7±1579.6	7729.3±2074.5
SM 34:0	4369±686.2	5402.1±1442.6	6326±803.1⁎⁎
SM 34:1	120736.7±12384.6	129323.9±26176.6	129129.6±22409.9
SM 34:2	15620.5±2496.8	15680.2±1294	16322.5±5561.5
SM 34:3	135.9±24.5	124.9±19	163.3±98.7
SM 35:1	4815.2±876.8	4845.4±1167.2	5248.6±1542.4
SM 35:2	773.9±190.4	705.4±185.2	781.8±310.9
SM 36:1	22607±3503.3	25721.4±3228.3	25610.4±5220.1
SM 36:2	9749.1±1996.5	9579.6±1292.4	10097.5±3951.9
SM 36:3	1074.4±360.1	924±190.8	1074±643.2
SM 37:2	512.7±192.1	500.2±173.8	521.5±291.6
SM 38:1	20644±4108.9	19264.3±2275.6	21268.4±5843.8
SM 38:2	6799.4±1206.8	6278.9±773.2	6563.5±1873.8
SM 39:1	10157.8±2045.2	7490.5±1875.6	7619.5±2037.8
SM 41:1	18996±4036.7	17979.3±2004.9	17306.7±6331.9
SM 41:2	19727.3±3049.4	17171.9±2962.8	16034.9±5041.4
SM 42:1	26934.6±4203.4	25312±5100	26693.5±8431
PC 28:0	368.6±286.4	376.4±250.1	435.5±168.8
PC 29:0	91.2±48.6	95.1±54.7	116.8±62.2
PC 30:0	4134.8±2489.4	4184.1±1746	4956.2±2064.6
PC 31:1	1389.3±208.7	1454.6±267.7	1555.7±508
PC 31:0	979.1±388.1	1054.1±406.6	1209.8±563.1
PC 32:3	458.5±86.5	376.4±85.7	421±155.2
PC 32:2	7219.7±1407.8	6448.7±1665.9	6654.3±2050.4
PC 32:1	29454.1±25941.6	37082.1±23455	30935.6±15626
PC 32:0	12671.1±2388.3	13403.8±2964.8	17114.9±6334
PC 33:3	5682.1±1553.1	4107.9±708.2	4656.7±1065.8
PC 33:2	4583.7±1419.7	3659.3±1057.2	3564.5±1192.7
PC 33:1	4384.5±1011.4	5046±1359.2	4481.8±1552
PC 33:0	1655.6±348.4	1631.8±314.1	1916.6±510.2
PC 34:5	171.6±70.9	229.6±186.5	240.4±98.6
PC 34:4	1606.8±581.8	1652.5±786.6	1834.3±717
PC 34:3	22769.1±7352	19858.9±5837.6	20107.6±5685.3
PC 34:2	321046.6±34684	290899.5±22001.8	293708.3±41432.2
PC 34:1	195585.8±52480.2	206850.7±55249.4	197388.1±53998.2
PC 34:0	4348.4±721.1	4120.7±721.3	5802.8±2909.4
PC 35:5	113.2±51.2	157.6±72.2	169.3±75.2
PC 35:4	1240.9±652.1	1203±432.6	1246.2±501.3
PC 35:3	1901.1±134.7	1581.4±501.9	1506.1±592.2
PC 35:2	13544.2±2490.1	9696.8±2595.7	10090.5±2772.5
PC 35:1	8526.9±1780.9	7819.8±1872	7446.2±1690.3
PC 35:0	320.7±67.9	281±72.2	391.4±120.7
PC 36:6	861.1±256.2	1095.5±981.7	1001.5±345.8
PC 36:5	25911.8±9200.7	27956.2±10197.1	38102.1±10058.6
PC(16:0/20:4)	112686.7±26551.2	121232.6±21119.6	127767.4±32566.9
PC(18:1/18:3)	112686.7±26551.2	121232.6±21119.6	127767.4±32566.9
PC 36:3	152274±15318.6	142470.1±19125.1	143953.6±22671
PC 36:2	252660.6±29711.9	210302±11360.1	218774.2±27341.8
PC 36:1	65109.3±34001.4	60096.2±11547.4	66580.9±27261.1
PC 36:0	406.3±101	301.9±56.4	791±632.5
PC 37:6	797.3±362.2	743.9±118.1	698±223.9
PC 37:5	997.2±204.1	1155±445	1190.7±384.4
PC 37:4	6743.4±2109.5	5567.9±1941.8	5887.4±1628.4
PC 38:7	2140.7±487.2	2072.9±1360.1	2200.9±673.7
PC(16:0/22:6)	51011.5±10081.2	53855.3±21311.4	52577.1±9403.7
PC(18:2/20:4)	7393.4±1510	6016.4±1803.3	6887.3±2773.6
PC 38:5	59549.9±14643	57159.2±11130.5	64488.3±12874.4
PC 38:4	114510.3±30187.9	103817.1±15881.4	117427.7±34441.3
PC 38:3	50030.3±10091.2	54515.2±17004.9	52342.7±17609.6
PC 38:2	34797.2±6120.1	33767.7±4012.4	33369.1±6714
PC 39:7	83±16.7	66.9±27.2	70.3±19.5
PC 39:6	2128±631.7	1710.6±254.6	1721±573
PC 39:5	1169.4±171.9	958.2±154.9	993.3±286.5
PC 40:8	1759.1±159.3	1433±347.5	1964.9±654.6
PC 40:7	6129.3±1808.9	4631.1±1161.6	5225.3±1183.4
PC 40:6	25202.8±5582.4	28273.7±8375.1	29250.9±13740.5
PC 40:5	19341.9±7261.4	18482.1±3718.4	21315±7927.1
PC 40:4	3645.4±1483.4	3399±698.9	4450.1±2350.6
PC 14:0_0:0	1450.5±967.4	1648.7±700.8	2068.5±1021.5
PC 15:0_0:0	712.4±188.1	909.3±398.6	862.4±378.9
PC 16:0_0:0	64035.6±16477.7	81270.5±24156.4	79525±28789.3
PC 16:1_0:0	3073.1±3006.7	3838.9±2309.4	3347.7±1738.2
PC 17:0_0:0	1733±304	1781.3±598	1721.4±591.5
PC 17:1_0:0	412.9±226.4	475.9±140.3	447.3±197.6
PC 18:0_0:0	20686.9±4892	22679±4473.8	24098.5±8511.9
PC 18:1_0:0	20484.1±12961.7	19506.7±7474.2	16096.6±5343.9
PC 18:2_0:0	25858.1±3718.1	20702.9±4240.8	16748.8±5693.6
PC 18:3_0:0	878.5±600.8	686.3±236.6	620.4±268.8
PC 20:0_0:0	140.6±47.7	108±20.6	160.9±74
PC 20:1_0:0	284.4±142.5	252.4±75.3	357.9±153.6
PC 20:2_0:0	161.7±74.9	136.2±54.8	155.3±60.2
PC 20:3_0:0	2678.8±1133.1	2945.5±1104.9	2085.1±1098.2
PC 20:4_0:0	5258.2±1461.7	5528.7±1435.1	4635.1±2121.9
PC 20:5_0:0	712.6±301.9	1214.2±1064.5	900.4±451
PC 22:0_0:0	33.8±4.5	25±5.2	28±10
PC 22:1_0:0	20.8±10.5	19.3±5.2	23.3±8.2
PC 22:5_0:0	693.9±404	642.1±211	561.9±224.4
PC 22:6_0:0	1521.4±489.9	1750.5±1071.8	1209.3±335.3
PC 24:0_0:0	73±10.6	55±17	56.3±11.4
PC 26:0_0:0	18.6±3.3	15.7±6.8	16.1±3.1
PC(O-32:0)	1760.1±422.5	1725.1±264.1	1765±369.2
PC(O-32:1)	536.6±129	495.5±180.3	476.9±140.8
PC(O-32:2)	99.9±43.6	112.8±77.3	126.3±67.5
PC(O-34:1)	4309.9±585.3	3993.5±518.9	4198.4±983
PC(O-34:2)	4254.6±1277.4	3382.8±360.2	3632±916.7
PC(O-34:3)	123.9±19.7	96.6±20.8	117.2±49
PC(O-34:4)	263±28.9	232.9±90.6	254.6±114.7
PC(O-35:4)	137.1±46.9	168.3±62.8	172.6±101
PC(O-36:0)	62.2±18.2	42.6±10.1	164.9±154.3
PC(O-36:1)	699.4±170.2	550.8±92	673±284.1
PC(O-36:2)	2607.4±615.2	1795.9±208.6	2113±596
PC(O-36:3)	4460.3±850.7	4223.1±270.7	4185.6±869.3
PC(O-36:4)	11578.9±3052.7	12290.3±1957.4	12383.1±3596.9
PC(O-36:5)	568.1±195.7	885.1±499.9	899.8±360.8
PC(O-38:4)	8185.4±2462.8	8238.8±809.6	8352.4±2172.1
PC(O-38:5)	10828.4±2246.1	10739.9±916.4	11138.4±2171.4
PC(O-40:5)	2945.4±478.9	2783.5±435.7	2689.5±647.2
PC(O-40:6)	2031.5±445.6	2113.1±407.9	2130.7±363.3
PC(O-40:7)	1698.3±431.3	1701.5±440	1721.2±362.3
PC(P-30:0)	122.5±56.5	107.8±36.8	116.1±18.3
PC(P-32:0)	1330.4±440.4	1312.3±230	1423±230.3
PC(P-32:1)	225±73.5	225.4±90.2	245.9±85.2
PC(P-34:1)	2383±276.4	2218.2±425.3	2474.3±417.2
PC(P-34:2)	5682.1±1553.1	4107.9±708.2	4656.7±1065.8
PC(P-34:3)	87.3±14.4	88.2±19.8	113±41.4
PC(P-36:2)	2427.2±675	1886.1±418.2	2214.6±479.6
PC(P-36:4)	7145.8±731	7776.4±1691	8379.8±2858
PC(P-36:5)	576.1±118.2	827.7±441.2	958.4±574.4
PC(P-38:4)	2954.2±435.3	3072.1±540.9	3339.6±1290.2
PC(P-38:5)	4598.9±761.8	5059.4±1125.2	5301.6±1374.1
PC(P-38:6)	872.6±173.2	1079.8±468.5	1059±319.5
PC(P-40:6)	482.8±143.1	585.9±260.4	559.9±156.8
PC(O-16:0/0:0)	313±86.8	389.6±82.1	491.2±207.2
PC(O-18:0/0:0)	102.6±27.3	107.9±24.6	163.9±87.8
PC(O-18:1/0:0)	229.9±89.1	246.8±49.7	361.3±151.8
PC(O-20:1/0:0)	16.7±7.6	13.7±3.9	17.9±8.6
PC(O-22:0/0:0)	42.2±11.5	42±7.8	36.3±5
PC(O-22:1/0:0)	36.1±16.4	28±8	23.6±7.3
PC(O-24:0/0:0)	83.9±27.9	82.3±21	79.2±6
PC(O-24:1/0:0)	120.2±62.2	94.7±27.1	85.2±13.8
PC(O-24:2/0:0)	20.8±8.5	11.4±4	13.2±2.9
PE 32:1	112.3±173.3	136.9±140.1	147.1±151.1
PE 34:1	1786.3±2028.9	1667.2±896.3	2553.2±2061.4
PE 34:2	2699.6±1330.1	2753.3±1212	3475.8±1896.7
PE 34:3	224.1±197.4	184.7±101.7	230.4±146.8
PE 35:1	161.8±146.1	136.7±60.1	170±89.9
PE 35:2	184.5±99.1	146.9±76.7	174.3±67.8
PE 36:1	2235.9±2702.6	1328.5±368	2849±2193.8
PE 36:2	9985.6±7296.1	6783.9±1663.9	11031.4±6448
PE 36:3	3153±2349.3	1952.6±598.7	3443.1±1787.9
PE 36:4	2838.8±1220.9	2853.6±1199.9	4855.5±3665.1
PE 36:5	293.3±277.9	367.5±392.9	414.9±270.7
PE 38:3	1769.5±1145.2	1402.2±612.9	2760.4±2211.3
PE 38:4	8155.7±3969.9	6235.9±1782.5	15795.8±13903.8
PE 38:5	3251.4±2184.1	2955±1455.2	5273.9±3989.3
PE 38:6	3358.7±1034.7	4436.2±2952.1	4777.5±3375.1
PE 40:4	231.2±212.1	129±40.9	776.9±873.6
PE 40:5	845±626.1	772.3±285.5	1573.2±1389
PE 40:6	1985.1±1012.5	2217.1±989.5	2780±2053.9
PE 40:7	390.9±251.1	314.6±173.2	537±386.4
PE(O-18:0/22:5)	189.5±38.7	201.9±68.7	224.1±97.6
PE(O-18:1/18:2)	170.6±46.1	173.6±71.8	134.4±45.7
PE(O-18:1/20:3)	576.3±102.3	646.5±208.9	851±409.8
PE(O-18:2/18:2)	600.3±127.1	762.9±221.5	734.7±287.9
PE(O-18:2/20:3)	823.9±73.2	926.8±336.4	887.4±303.9
PE(O-18:2/22:5)	213.8±28.5	226±92.4	241.5±71.4
PE(O-34:1)	152.2±31.8	147.1±67.8	139.8±41.8
PE(O-34:2)	92.1±35.9	89.4±35.5	78±32.8
PE(O-36:2)	70.5±16.7	88.7±39.9	86±39.5
PE(O-36:5)	65.7±14	93.1±72.3	91.4±65.1
PE(O-36:6)	34.3±10.4	71.7±44.9	95.4±32.5⁎⁎
PE(O-40:6)	210.2±24.8	210.4±52.8	208.9±86.2
PE(P-16:0/18:1)	66.7±12.8	84.4±38.6	107.8±60.8
PE(P-16:0/18:2)	100.6±40.4	135.4±50.3	127.9±27.1
PE(P-16:0/20:4)	331.1±19.9	409.4±122.2	951.1±754.5
PE(P-16:0/22:5)	1032.9±105.8	1382.6±732.5	1628±691.8
PE(P-16:0/22:6)	273.1±59.2	353.6±172.7	374.1±76.2
PE(P-18:0/18:1)	70.5±16.7	88.7±39.9	86±39.5
PE(P-18:0/18:2)	311.5±118.8	337.1±104.9	329.7±73.2
PE(P-18:0/20:4)	737±81	799.3±260.2	1987.7±1556.7
PE(P-18:0/22:5)	395.8±50.8	449.6±258.8	627.6±322.6
PE(P-18:0/22:6)	219.4±36.2	286.2±197.7	286.2±77.5
PE(16:0_0:0)	1355.4±601.2	1827.6±933.3	1669.1±711.2
PE(18:0_0:0)	2434.3±1267.8	2559.4±852	3768.1±2358.8
PE(18:1_0:0)	3437.2±3836.1	2205.6±691.9	2285±847.4
PE(18:2_0:0)	4242±2115	3335.9±937.7	2380.2±564.1
PE(20:4_0:0)	1891.9±667.5	1702.2±298.4	1576.4±452
PE(22:6_0:0)	1377.9±402.5	1723.5±836.7	1042.7±168.4##
PI 32:0	384.5±238.9	1302.4±1204.3	1240.2±1080.8
PI 32:1	819.7±771.7	3314.5±3854.2	2080.4±1952.6
PI 34:0	153.5±58	362.2±298.8⁎	342.8±287.7
PI 34:1	8427±4815.1	17398.1±11562.1	12850.4±9325.1
PI 36:1	9148.6±4428.3	11986.8±4399.9	9382.8±4817.2
PI 36:2	22965.5±6974.4	25557.5±8566.8	20820±3299.5
PI 36:3	6773.6±3294.2	6093.6±1601.8	6415.4±1885.4
PI 36:4	6193.6±2762.2	6555.4±2189.8	28562.8±50448.9
PI 38:2	775.6±536	540±103.1	738.7±412.5
PI 38:3	11259.1±4650.4	8023.3±1480.1	9582.2±2559.5
PI 38:4	42280.7±11985.9	28710.8±5496.6	47736.2±21064.5
PI 38:5	4390.8±3078.9	3827.9±1264.1	5561.7±2560.2
PI 38:6	901.9±428.9	1641.8±1327.5	1364.7±561.9
PI 40:4	559.6±250.2	515.2±62.7	804.8±532.7
PI 40:5	1608.8±583.2	2479±935.9	2218.6±988.7
PI 40:6	1647±616.1	2981.7±2190.1	2165.5±928.8
PI (18:0_0:0)	707.4±218.5	700.5±143.1	1378.7±872.5
PI (18:1_0:0)	490.2±206.2	710.8±385.3	634.2±284.8
PI (18:2_0:0)	495.9±156.9	692.2±365.4	494.9±192.9
PI (20:4_0:0)	650.2±161.5	589±126.1	629.7±142.3
PS 36:1	1418.5±935.8⁎⁎	259.4±259.9	7733.4±9425.3#
PS 38:3	299.8±158.4⁎	53.7±91.9	1559.3±1791.7#
PS 38:4	191.7±133.1	135.7±62.2	316.2±244.8
PS 40:5	123±65.4⁎⁎	13.6±33.4	590.9±659.3#
PS 40:6	128.3±53.9	36.5±49.6	572.1±619.6#
PG 18:0_18:1	110.6±101.9	76.4±26.9	105.7±68.8
PG 18:1_18:1	80.4±71.6	66.1±26.6	88.1±55.1
CE 14:0	19911.2±15468.1	21884.7±9734.9	23811.5±9856.2
CE 15:0	17699.3±5671.7	22221.3±10818	20997.5±6853.8
CE 16:0	318876.5±42174.4	365155.6±30084.7	353655.5±52830.9
CE 16:1	115561.6±152024.7	151872.5±70309.1	134746.3±92283.7
CE 16:2	2779.4±1580.8	3445.8±1357.5	3464.8±1066.5
CE 17:0	8220.8±1483.9	9469.3±4026.2	7904.5±2728.8
CE 17:1	34230.6±8103	42325.3±9076.7	38586.9±7584.2
CE 18:0	17830.9±2511.8	20183.7±6264.9	15798.2±5758.3
CE 18:1	300937±93437.4	304312.4±43611.3	281342.1±59416.8
CE 18:2	318759.8±45580.4	350004.5±54887	329663.9±68020.6
CE 18:3	53088.2±26201.8	57313.5±16055.8	54718.4±19826.1
CE 20:1	272.6±60.2	255.9±71.9	225.5±45.5
CE 20:2	1476.3±554.3	1848.2±545.9	1611.8±644.6
CE 20:3	21197.7±6332.2	22717.8±4660	21939.8±7333.3
CE 20:4	125537.8±42656.5	145713.5±27527.5	139683.5±54248.4
CE 20:5	34335.7±15700.4	49320.2±11003	49395±20742.3
CE 22:0	185.1±43.8	181.4±66.6	154.1±65.3
CE 22:1	94.4±35	93.7±39.9	80.5±22.7
CE 22:4	61.6±29.4	72±18.9	72±20.6
CE 22:5	1013.3±410.8	1045.9±225.5	1056.1±331.8
CE 22:6	14640.6±4365.5	17865.2±3604.4	15897.9±6664.7
CE 24:0	157.1±66.7	166.1±56.8	141.1±58.4
CE 24:1	151.5±67.3	130.3±40.6	117.7±30
CE 24:5	11.2±4.5	9.9±4.5	11.9±7.6
CE 24:6	13.3±13.7	11.9±4.1	12.6±8.7
COH	919851.6±43495	886588.6±126000.8	878693.3±260061.4
DG 14:0_14:0	24.5±29.2	28±21.7	80.6±110.5
DG 14:0_16:0	458.3±462.2	623.8±328.4	1302±1164.1
DG 14:0_18:1	1160.6±1314.6	1325.1±604	2139.1±1769.8
DG 14:0_18:2	369.9±197.1	434.6±274.5	666.6±555.3
DG 14:1_16:0	59.7±93.9	66.5±41	126.9±97.4
DG 16:0_18:1	7495±5862.4	10715.9±3041.9	17638.8±13327.8
DG 16:0_18:2	2318±916.1	2589.8±669.2	4784.2±3174.3
DG 16:0_20:0	180.1±113.4	174.5±34.5	307.6±147.6
DG 16:0_20:3	162.1±165.4	240.9±99.2	401.8±299.6
DG 16:0_20:4	388.7±311.5	663.2±306.4	1051.5±898.8
DG 16:0_22:5	190.4±45.8	281.5±126.8	422.3±221.5
DG 16:0_22:6	203.2±132.7	284±122.7	552.8±376.6
DG 16:1_18:1	3118.2±3175.2	3348.5±1197.4	4167.9±2343.6
DG 18:0_16:1	217.8±339.7	272.2±142.2	450.2±423.4
DG 18:0_18:1	1744.2±1866.3	2014.4±596.2	3535.7±3186.2
DG 18:0_18:2	824.4±434.3	933.8±322.2	1616.1±1457.3
DG 18:0_20:4	252.9±148.4	240.2±49.4	605.5±397.4
DG 18:1_18:1	14549.8±9201.7	13502.3±4060.8	17117.7±9597.7
DG 18:1_18:2	10383±4910.3	7497.6±2434.9	10707.9±6581.3
DG 18:1_18:3	1980.3±1524.4	1303.2±480.4	2004.9±1623.8
DG 18:1_20:3	831.6±581.4	681.8±211.2	943.7±361.1
DG 18:1_20:4	2052.9±1339.5	2009.6±658.1	2734.6±2134.5
DG 18:2_18:2	2051.3±1172.5	1121.7±505.6	1846.7±1348.7
TG 14:0_16:0_18:2	2119.9±3113.8	1681.5±824.2	1786.2±981
TG 14:0_16:1_18:1	15689.1±24325.8	12817±6682.9	16676±10702.5
TG 14:0_16:1_18:2	2356.8±2863.1	1815.6±1350.6	2527±2157
TG 14:0_18:0_18:1	538.5±552.8	460.9±131.6	462.6±218
TG 14:0_18:2_18:2	1137.3±518.5	713.1±465	962.1±755.1
TG 14:1_16:0_18:1	2913.9±4586.8	3061.4±1774.1	3288.7±2061
TG 14:1_16:1_18:0	11433.7±18825.8	12185.2±6784.1	13214.2±11740.7
TG 14:1_18:0_18:2	587.9±814	367.3±145.7	402.8±254.7
TG 14:1_18:1_18:1	5765.4±5038.3	4539.6±1635	4974.2±2103.2
TG 15:0_18:1_16:0	2349.5±2154.4	3097±1687.9	3683.8±2197.4
TG 15:0_18:1_18:1	787.1±219.4	906.1±477.9	802.7±381.6
TG 16:0_16:0_16:0	2670±2206.5	4654.4±2248.9	6093.1±4616.8
TG 16:0_16:0_18:0	5670.6±3855.1	7917.6±2955.5	11025.9±7699.3
TG 16:0_16:0_18:1	39184.4±32509.9	56361.6±19969.8	65983.9±39537.3
TG 16:0_16:0_18:2	7858.6±3073.8	10895.8±4724.1	12934.1±6843.3
TG 16:0_16:1_18:1	53777.2±54131.9	58534.3±20253.6	60182.4±28638.4
TG 16:0_18:0_18:1	11200.2±12018.9	14306.1±5315.3	16854.7±12231.3
TG 16:0_18:1_18:1	96078.5±49303.2	102766.9±29150.6	94045.1±30306.6
TG 16:0_18:1_18:2	50245.6±14957.3	44603.8±14388.1	45817±19032.6
TG 16:0_18:2_18:2	10728.3±4558.1	7949.2±3049.6	9207.8±4769.3
TG 16:1_16:1_16:1	1298±1974.5	978.3±481.6	1075.6±613.7
TG 16:1_16:1_18:0	1118.1±1548.6	1097.3±591.6	1628.9±1386.9
TG 16:1_16:1_18:1	5830.4±6020.4	6054±2336.5	5832.5±2611.2
TG 16:1_18:1_18:1	8334±6440.3	6584±2595.6	5665.5±1726.4
TG 16:1_18:1_18:2	16271.1±8921.3	11648.1±3692.4	12053.5±4609.8
TG 17:0_16:0_16:1	412.6±119.6	578±194.7	437.3±165.9
TG 17:0_16:0_18:0	33.1±29.6	45.2±24.4	38.3±15.6
TG 17:0_18:1_14:0	256.1±94.5	386.3±119.9	280.7±154.7
TG 17:0_18:1_16:0	1904.5±1106.6	2861.4±1753.5	2842.9±1672.3
TG 17:0_18:1_16:1	282.5±66.4	345.2±108.8	231.7±71.5
TG 17:0_18:1_18:1	3862.7±1563	4070.7±1405.4	3657.8±1141
TG 17:0_18:2_16:0	277.6±60.9	310.2±81.5	223.6±53.8
TG 18:0_18:0_18:1	1530.8±2332.7	1447.7±702.3	2256.5±2571.5
TG 18:0_18:1_18:1	13343.8±13301.1	10862.7±3685.2	12069.8±8074.6
TG 18:0_18:2_18:2	3080.7±1786.8	2469.2±1537	2553.7±1934.8
TG 18:1_14:0_16:0	9937.5±11568.4	11871.3±6719.1	15809±10286.6
TG 18:1_18:1_18:1	26949.1±22999.8	16927±6518.7	16775.2±8249.9
TG 18:1_18:1_18:2	10502.4±7340.7	5232.1±2012.8	6180.4±4024.4
TG 18:1_18:1_20:4	1208.6±674.2	1026.1±284	1080.7±620.9
TG 18:1_18:1_22:6	1655.5±877.7	1705.9±490.9	1594.5±831
TG 18:1_18:2_18:2	11330.7±10454.7	4571.6±1798.6	5623±4646.3
TG 18:2_18:2_18:2	1150.9±1011.2	401±172.2	558.7±526.4
TG 18:2_18:2_20:4	294.9±212.7	212.5±88.1	285.4±253.6

Abbreviations: dhCer, dihydroceramide; CE, cholesteryl ester; Cer, ceramide; COH, cholesterol; DG, diacylglycerol; DHC, dihexosylceramide; GM3, G_M3_ ganglioside; MHC, monohexosylceramide; PC, phosphatidylcholine; PC(O), alkylphosphatidylcholine; PC(P), alkenylphosphatidylcholine; PE, phosphatidylethanolamine; PE(O), alkylphosphatidylethanolamine; PE(P), alkenylphosphatidylethanolamine; PG, phosphatidylglycerol; PI, phosphatidylinositol; PS, phosphatidylserine; SM, sphingomyelin; TG, triacylglycerol; THC, trihexosylceramide.

Means ± SD are shown. Concentrations are given in pmol/ml.

⁎ *p*<0.05 vs HIV Negative.

⁎⁎ *p*<0.01 vs HIV Negative.

# *p*<0.05 vs WT HIV.

## *p*<0.01 vs WT HIV (ANOVA).
